# Sex-specific effect of dietary fatty acids on non-alcoholic fatty liver disease

**DOI:** 10.3389/fnut.2025.1582527

**Published:** 2025-07-02

**Authors:** Jianhua Chen, Zeqin Zhang, Yuning Pan, Jiejun Shi

**Affiliations:** ^1^Department of Radiology, The First Affiliated Hospital of Ningbo University, Ningbo, Zhejiang, China; ^2^Department of Cardiology, The First Affiliated Hospital of Ningbo University, Ningbo, Zhejiang, China; ^3^Department of Infectious Diseases, The First Affiliated Hospital of Ningbo University, Ningbo, Zhejiang, China

**Keywords:** non-alcoholic fatty liver disease, fatty acids, steatosis, sex, age

## Abstract

**Background:**

Non-alcoholic fatty liver disease is the most common chronic hepatic disease worldwide. Dietary fatty acid is tightly associated with the development of non-alcoholic fatty liver disease but few large-scale and in-depth clinical researches have focused on the issue.

**Methods:**

We conducted a retrospective case-control study based on the data from the 2017–2018 cycle of the National Health and Nutrition Examination Survey.

**Results:**

A total of 2,470 adult participants were included in this study. Logistic regression analysis showed that dietary fatty acids were positively associated with non-alcoholic fatty liver disease (odds ratio and 95% confidence interval >1 and *P* < 0.05) except for polyunsaturated fatty acid. Subgroup analysis stratified by age stage and weight grade revealed that aforementioned association was significant only in individuals aged group 37–55 and those classified as obesity. In addition, all the fatty acid related ratios (the ratio of unsaturated fatty acids to saturated fatty acids, the ratio of polyunsaturated fatty acid to saturated fatty acid, the ratio of monounsaturated fatty acids to saturated fatty acids, the ratio of polyunsaturated fatty acids to monounsaturated fatty acids) showed protective effects against the onset and steatosis severity of non-alcoholic fatty liver disease in men, as evidenced by stratified logistic regression analysis (all the odds ratio [95% confidence interval] < 1 and *P* < 0.05) and smooth curve fittings.

**Conclusion:**

These findings suggest that dietary fatty acids modification could serve as a preventive strategy for male non-alcoholic fatty liver disease. Increasing the proportion of unsaturated fatty acids in the diet, especially polyunsaturated fatty acids, is promising to prevent non-alcoholic fatty liver disease in middle-aged obese men.

## Introduction

Non-alcoholic fatty liver disease (NAFLD) is the most common chronic liver disease, affecting approximately 25% of the global population (1). It is characterized by excessive intrahepatic lipid accumulation, oxidative stress, and inflammation. Around 3%−5% of NAFLD patients may develop non-alcoholic steatohepatitis (NASH) and have a higher risk for liver failure, cirrhosis and hepatocellular carcinoma (1). Although a series of lipid metabolisms occur in the liver, liver is not designed for lipid storage (2). Disturbance of hepatic lipid metabolism and lipotoxicity play a key role in the pathophysiology of NAFLD (1). As one of the main sources of lipids, diet is well established to have a tight relationship with NAFLD, though the underlying mechanisms are not fully understood. Numerous reviews have highlighted the deleterious effect of saturated fatty acids and the protective role of unsaturated fatty acids in the development of NAFLD (3–9). Despite these insights, there is currently no definitive therapeutic medication for NAFLD in clinical practice, making lifestyle and dietary modifications crucial for prevention (6). There were some small-scale prospective studies attempting to confirm the therapeutic potential of unsaturated fatty acids on NAFLD (10, 11), nevertheless large-scale prospective and retrospective clinical studies focusing on the association between different types of fatty acids and NAFLD are still lacking. Although most literature supported that polyunsaturated fatty acids (PUFA) could reduce the risk of NAFLD (11–13), some animal experiments indicated that PUFA aggravated liver damage in mice with NAFLD (14), which means the influence of PFUA on NAFLD remains controversial. Moreover, the role of sex, age, and body weight on the relationship between dietary fatty acid intake and NAFLD has not been thoroughly investigated. Herein, we conducted this study to explore and compare the impacts of various fatty acids and related ratios on NAFLD by analyzing large-scale retrospective data from National Health and Nutrition Examination Survey (NHANES) database.

## Methods

### Study sample

**Figure 1:**
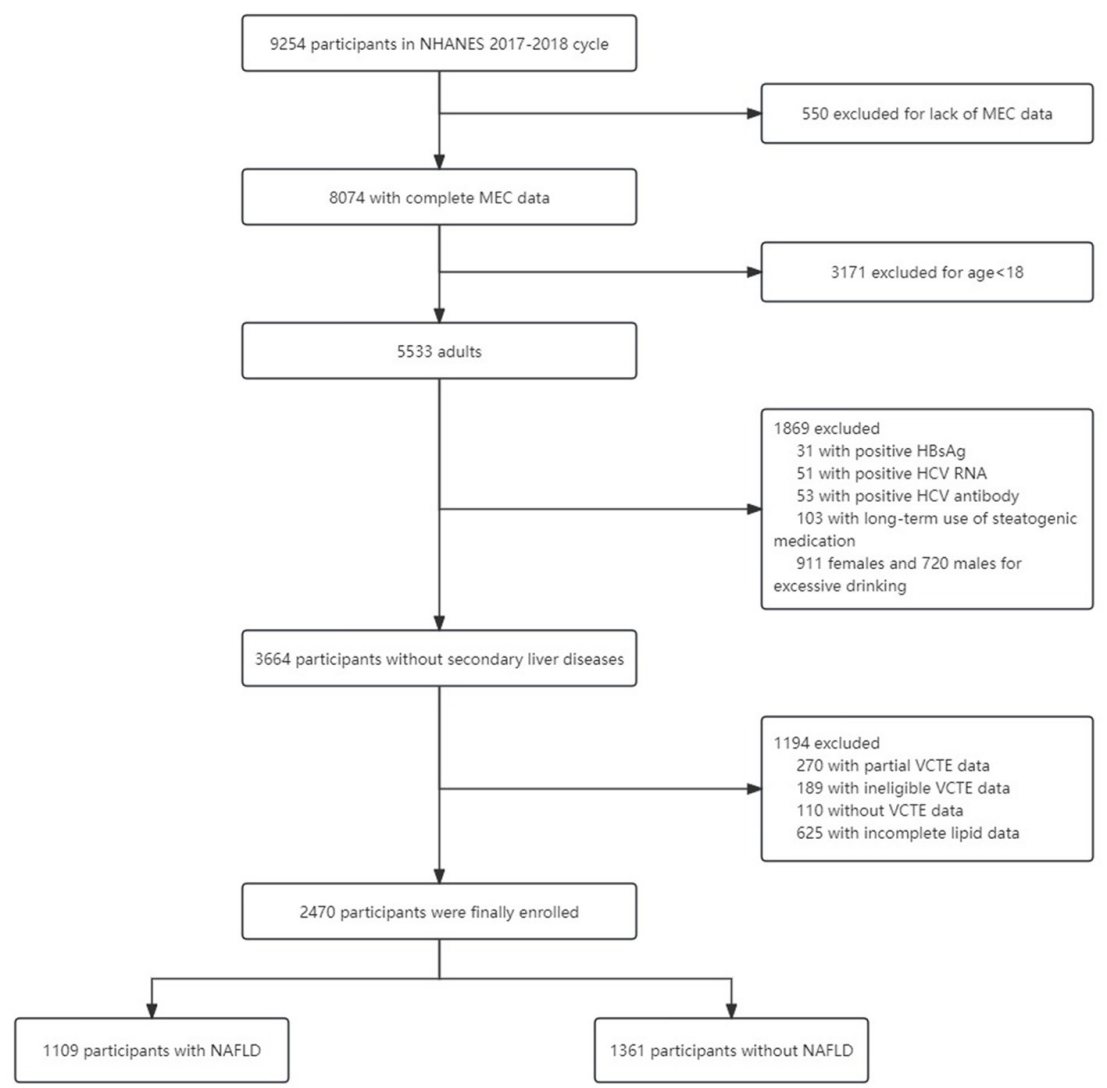
The flowchart of data cleaning.

We analyzed the data from the 2017–2018 cycle of the NHANES which is a nationally representative survey of nutrition and health condition in the United States. The whole survey consists of two parts: a structured interview performed at home and a standardized health checkup at a mobile examination center (MEC). The detailed eligibility criteria for participant enrollment are described in Figure 1. Finally, 2,470 participants with eligible data were included.

### Study variables and definitions

Demographic data, including age, sex, and race, were obtained from demographic questionnaires. The dietary components were acquired from interviews on the dietary recall of the past 48 h. The intake of various nutrients was estimated via the average daily intake value. Height (cm), weight (kg), and waist circumference (cm) were measured by NHANES staff during the MEC visit. Body mass index (BMI) was calculated as weight in kilograms divided by height in meters squared. Weight grades were categorized as normal weight, overweight and obesity based on BMI values. BMI < 25 kg/m^2^ was regarded as normal weight, overweight was defined as BMI of 25–29.9 kg/m^2^, and obesity was confirmed when BMI ≥ 30 kg/m^2^ (15). Diabetes was diagnosed with any of the following conditions (16): (1) Fasting plasma glucose (FPG) ≥126 mg/dl (7 mmol/L); (2) Random plasma glucose ≥200 mg/dl (11.1 mmol/L); (3) glycohemoglobin (HbA1c) level ≥6.5% (48 mmol/mol); (4) Self-reported diabetes; (5) Using antidiabetic medicines. Hypertension was diagnosed as blood pressure of at least two office visits ≥140/90 mm Hg (17). Excessive alcohol intake was defined as >2 drinks per day for females and >3 drinks per day for males (18). Steatogenic medication includes corticosteroids, valproate, amiodarone, tamoxifen, methotrexate and antiretroviral drugs (18). NAFLD was confirmed when both of the following conditions were met: (1) controlled attenuation parameter (CAP) value ≥ 274 dB/m on vibration-controlled transient elastography (19). (2) Lack of secondary factors for hepatic steatosis as listed in Figure 1. Fibrosis stages were defined by median liver stiffness measurements (LSM) value: normal (F0) as LSM < 8.0 kPa, fibrotic non-alcoholic steatohepatitis (F2) as 8.0 to <12.0 kPa, advanced fibrosis (F3) as 12.0 to <20.0 kPa, and cirrhosis (F4) as ≥20 kPa (20). The methodology of laboratory tests, such as standard biochemistry profile, HbA1c, and serum lipid level, can be available on the NHANES website: https://wwwn.cdc.gov/nchs/nhanes/continuousnhanes/labmethods.aspx?BeginYear=2017.

The definitions of some key indices: RUS is short for the ratio of unsaturated fatty acids to saturated fatty acids. RPS is short for the ratio of polyunsaturated fatty acid to saturated fatty acid. RPM is short for the ratio of polyunsaturated fatty acids to monounsaturated fatty acids. RMS is short for the ratio of monounsaturated fatty acids to saturated fatty acids.

### Statistical analysis

Since the survey design was complex, stratified, multistage probability cluster sampling, weighted analysis was performed using the appropriate subsample weights, strata, and primary sampling units to eliminate potential bias as recommended by the National Center for Health Statistics. Software R (R-4.3.1 for Windows), SPSS Statistics 25 software (IBM, Armonk, NY, USA), EmpowerStats4.0 (https://www.empowerstats.net/en/), and GraphPad Prism 8.0.1 and Microsoft Excel were used to analyze data and build charts or figures. Categorical variables were expressed as weighted percentages (Sampling Error) and weighted mean (Sampling Error) for continuous variables.

The multivariable logistic regression analysis was used to assess the relationship between fatty acids, related ratios and NAFLD. The confounding factor was defined as interference on both the independent and dependent variables, but it's not their causal intermediate variable (21). Adjusted logistic regression models were constructed via adjustment of corresponding confounders according to different independent variables. Mediators such as BMI, blood lipid level, weight, etc. are not suitable to be regarded as covariates in the logistic regression analysis due to the mediating effect. The subgroup analysis is used to eliminate the bias caused by the interaction effect between sex and partial independent variables (22). Subgroup logistic regression analysis stratified by age stage and weight grade was conducted to explore the association of dietary fatty acid and NAFLD at each age stage and weight grade. To demonstrate more intuitive and comparable differences in the forest plot, dietary lipid components were converted into categorical variables based on their quartiles. Q1: ≤ 1st quartile; Q2: > 1st quartile and ≤ median; Q3: > median and ≤ 3rd quartile, and Q4: > 3rd quartile. The lowest quartile (Q1) was defined as the reference. Smooth curve fittings were performed to evaluate the non-linear relationship. Sampling design complexity is taken into account in all analyses. *P* < 0.05 was regarded as a statistically significant difference.

### Ethics declaration

The ethics review board of the National Center for Health Statistics approved all the NHANES protocols and all the participants have joined the survey with informed consent.

## Results

In total, 2,470 participants were included in our study among which 1,109 were diagnosed with NAFLD. Participants were divided into two groups: NAFLD and non-NAFLD. The weighted characteristics of the study subjects are summarized in Table 1. There were statistically significant differences between the two groups in demographic features (sex, age, race), dietary lipid intake [total fat (TTFAT), total monounsaturated fatty acids (TMUFA), and total saturated fatty acids (TSFA)], BMI, weight grade, waist circumference, hypertension, diabetes, current smoking state, HbA1c, liver function indexes (alanine transaminase, alkaline phosphatase, gamma-glutamyl transferase), uric acid, serum lipid (high-density lipoprotein cholesterol, triglyceride), FPG, current smoking state, CAP, and LSM values (*P* < 0.05). No significant difference was observed in total cholesterol, PFUA, low-density lipoprotein cholesterol or aspartate aminotransferase levels (*P* > 0.05). NAFLD was more prevalent in middle-aged and older men (*P* < 0.05), and the proportion of Mexican American individuals was notably higher in the NAFLD group than that in the non-NAFLD group (9.6% vs. 4.9%, *P* < 0.0001). Compared with non-NAFLD, participants with NAFLD exhibited worse metabolic indicators, including serum lipid, FPG, HbA1c, BMI, uric acid, and waist circumference, as well as higher prevalence of metabolic complications such as hypertension and diabetes, higher alanine transaminase level, more severe steatosis and fibrosis judged by CAP and LSM values. With regard to dietary lipid intake, TTFAT, TMUFA, and TSFA were significantly higher in the NAFLD group compared to the non-NAFLD group (*P* < 0.05).

**Table 1:** Weighted characteristics of participants grouped by NAFLD state.

**Item**	**Non-NAFLD**	**NAFLD**	***P* value**
**Sex**			<0.0001
Male	49.4 (1.5)	56.7 (2.1)	0.0011
Female	50.6 (1.5)	43.3 (2.1)	0.0011
**Age (years old)**	47.4 (0.7)	54.2 (0.6)	<0.0001
18–36	35.2 (2.2)	16.3 (1.2)	<0.0001
37–54	25.3 (2.1)	27.9 (2.7)	0.4662
55–66	21.8 (1.6)	30.5 (2)	0.0027
67–80	17.6 (1)	25.3 (1.6)	0.0002
**Race**			0.0003
Mexican American	4.9 (0.9)	9.6 (1.9)	<0.0001
Other Hispanic	6.4 (1.1)	5.3 (0.9)	0.3199
Non-Hispanic White	64.0 (2.9)	65.0 (3.3)	0.7482
Non-Hispanic Black	13.7 (1.8)	9.5 (1.6)	0.0018
Non-Hispanic Asian	6.7 (1)	6.3 (1.2)	0.7111
Other Race - Including Multi-Racial	4.2 (1.2)	4.3 (1)	0.9326
**Total fat (gm) and ratios**	82.4 (1.3)	88.7 (1.7)	0.0058
Total saturated fatty acids	26.2 (0.5)	29.2 (0.7)	0.0029
Total monounsaturated fatty acids	28.5 (0.5)	30.6 (0.6)	0.0017
Total polyunsaturated fatty acids	19.7 (0.4)	20.3 (0.4)	0.3911
RPM	0.7 (0)	0.7 (0)	0.2805
RPS	0.8 (0)	0.8 (0)	0.0842
RUS	1.9 (0)	1.8 (0)	0.0473
RMS	1.1 (0)	1.1 (0)	0.0578
**BMI (kg/m** ^**2**^ **)**	26.6(0.3)	33.3(0.5)	<0.0001
**Weight grade**			<0.0001
Normal weight	42.0 (2.4)	6.8 (1.4)	<0.0001
Overweight	35.3 (1.8)	26.7 (3)	0.0257
Obesity	22.7 (2.2)	66.5 (3.8)	<0.0001
**Waist circumference (cm)**	92.9 (0.8)	110.8 (1.1)	<0.0001
**Hypertension**	29.3 (2.1)	56.5 (3.2)	<0.0001
**Diabetes**	7.0 (0.8)	29.6 (2)	<0.0001
**Smoking**	30.0 (3)	20.2 (2.6)	0.0227
**HbAlc (%)**	5.5 (0)	6.1 (0)	<0.0001
**ALT (U/L)**	19.2 (0.5)	25.5 (0.6)	<0.0001
**AST (U/L)**	20.7 (0.5)	22.0 (0.4)	0.1115
**ALP (IU/L)**	74.3 (1.3)	79.9 (1.3)	0.0073
**GGT (IU/L)**	21.8 (0.8)	31.3 (1)	<0.0001
**Uric acid (mg/dL)**	5.3 (0.1)	5.8 (0)	<0.0001
**HDL-c (mg/dL)**	55.2 (0.8)	48.2 (0.8)	<0.0001
LDL-c (mg/dL)^*^	112.9 (2.3)	110.7 (2.8)	0.4342
**Triglyceride (mg/dL)**	90.3 (1.9)	147.2 (8.9)	<0.0001
**Total cholesterol (mg/dL)**	186.4 (2.9)	186.2 (3.3)	0.9527
**Fasting glucose (mg/dL)**	103.9 (0.9)	124.6 (3.6)	0.0001
**LSM (kPa)**	4.9 (0.1)	6.5 (0.2)	<0.0001
**CAP (dB/m)**	220.3 (1.7)	322.4 (2.2)	<0.0001
**Fibrosis level**			<0.0001
F0	96.1 (0.9)	84.0 (1.8)	<0.0001
F2	2.8 (0.9)	10.1 (1.4)	0.0056
F3	0.7 (0.3)	3.7 (1)	0.001
F4	0.4 (0.2)	2.3 (0.7)	0.0135

Smoking: current smoking state;

* LDL-Cholesterol, Friedewald. Wtsaf4yr was used for weighted analysis of fasting items while Wtmec2yr was used for other items. Survey-weighted linear regression was used to calculate the *P* values of continuous variables. Survey-weighted Chi-square test was used to calculate *P* values of categorical variables.

For continuous variables, data were presented as survey-weighted mean (sampling error). For categorical variables, data were presented as survey-weighted percentage (sampling error).

RUS, the ratio of unsaturated fatty acids to saturated fatty acids; RPS, the ratio of polyunsaturated fatty acid to saturated fatty acid; RPM, the ratio of polyunsaturated fatty acids to monounsaturated fatty acids; RMS, the ratio of monounsaturated fatty acids to saturated fatty acids.

**Figure 2:**
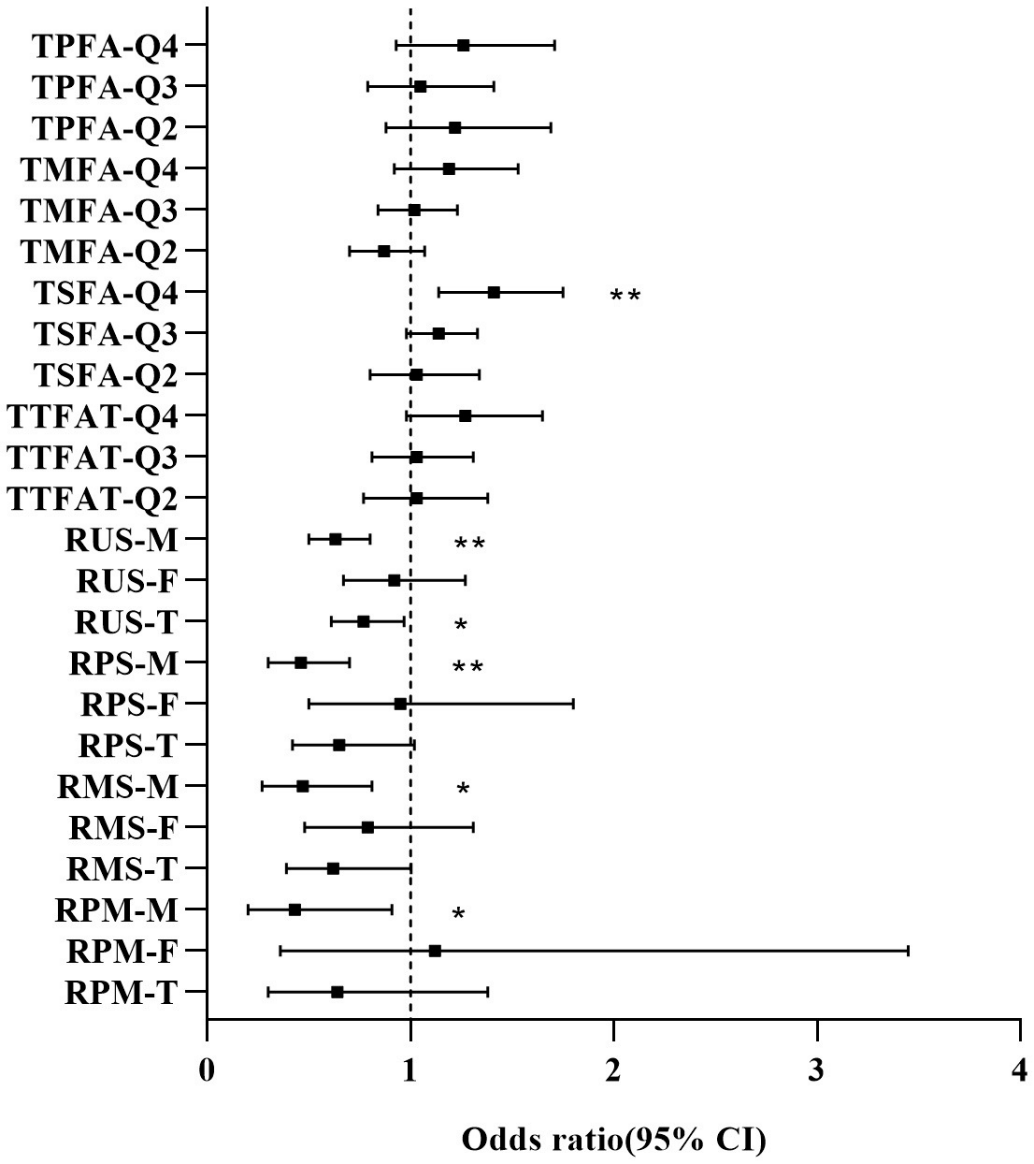
Forest plot for OR [95% CI] of dietary fatty acids and related ratios on NAFLD. M, Male; F, Female; T, Total; Q, Quartile. OR and 95% confidence interval were derived from weighted logistic regression without adjustment of covariates. ^*^P < 0.05, ^**^P < 0.01.

We further explored the dietary fatty acid intake and related ratios for their relationships with NAFLD via weighted multivariable logistic regression (Table 2). In the unadjusted Model 1, a significant positive association was observed between TTFAT, TMUFA, TSFA, and NAFLD [all the odds ratio (OR) and 95% confidence interval (CI) > 1, *P* < 0.05], indicating that higher intake of these lipids may increase NAFLD risk. Subgroup analysis stratified by sex showed the association only significant for TSFA in males. Then, we used univariate regression analysis to confirm confounding factors corresponding to each independent variable. In Model 2, which was adjusted for confounders, the significant positive association still survived between TSFA, TMSA and NAFLD. In terms of the ratio of each two kinds of fatty acids, Model 1 showed that only the ratio of unsaturated fatty acids to saturated fatty acids (RUS) was significantly associated with NAFLD. Since statistically significant interactions were verified between sex and the ratio of polyunsaturated fatty acid to saturated fatty acid (RPS), RUS, the ratio of polyunsaturated fatty acids to monounsaturated fatty acids (RPM) for NAFLD (*P* for interaction < 0.05), further logistic regression analysis stratified by sex on the ratios and NAFLD was performed. In the subgroup analysis, the situation was remarkably changed, significant associations between RPS, RUS, the ratio of monounsaturated fatty acids to saturated fatty acids (RMS), RPM and NAFLD were observed in males rather than females (*P* < 0.05). In detail, both unadjusted and adjusted ORs for NAFLD across the ratios were below 1 suggesting that higher ratios were linked to a lower likelihood of NAFLD in men. These associations remained significant in Model 2. Given the closeness of the ORs to 1, dietary lipid components (TTFAT, TSFA, TPFUA, and TMUFA) were categorized into quartiles for further analysis (Figure 2). The forest plot visually demonstrated whether the CI included 1. As shown in Figure 2, participants in the highest quartile of TSFA intake (Q4) had a noticeably higher risk of NAFLD compared with those in the lowest quartile (Q1) (OR [95% CI] > 1, *P* < 0.01). Additionally, we conducted a weighted logistic regression analysis of dietary lipid components on NAFLD stratified by age stage (divided by quartiles) and weight grade (Table 3). TTFAT, TSFA, and TMUFA were positively correlated with NAFLD in participants 37–54 years old (all OR [95%CI] > 1 and *P* < 0.05). Notably, this age stage marked a turning point. In participants younger than 37 years old, non-NAFLD is significantly more prevalent than NAFLD while in participants over 54 years old, NAFLD is dominant (Table 1). Subgroup analysis stratified by weight grade showed that OR values of TTFAT, TSFA, and TMUFA for NAFLD were significant only in obese individuals (OR [95% CI] > 1, *P* < 0.05). We further compared dietary lipid components and ratios between different sexes and discovered that intake of each dietary lipid component was significantly higher in males than females (*P* < 0.001) while insignificant differences were observed in most ratios between sexes (Table 4).

**Table 2:** Weighted logistic regression of association between fatty acids, ratios and NAFLD stratified by sex.

**Item**	**Subgroup**	**OR (95% CI) for Model1**	**OR (95% CI) for Model2**
TTFAT	Male	1.0027 (0.9986, 1.0068)	1.0024 (0.9972, 1.0077)
	Female	1.0041 (0.9996, 1.0085)	1.0041 (0.999, 1.0091)
	Total	1.004 (1.0015, 1.0065)^**^	1.003 (0.99996, 1.006)
TSFA	Male	1.014 (1.001, 1.028)^*^	1.0139 (0.9997, 1.0283)
	Female	1.012 (0.998, 1.027)	1.0124 (0.996, 1.0292)
	Total	1.0156 (1.0065, 1.0248)^**^	1.0133 (1.0003, 1.0265)^*^
TMUFA	Male	1.006 (0.996, 1.016)	1.0056 (0.9948, 1.0164)
	Female	1.009 (0.998, 1.02)	1.0092 (0.9969, 1.0217)
	Total	1.0095 (1.0046, 1.0144)^**^	1.0068 (1.0011, 1.0126)^*^
TPUFA	Male	0.9953 (0.9818, 1.0089)	0.994 (0.977, 1.011)
	Female	1.013 (0.994, 1.033)	1.014 (0.994, 1.034)
	Total	1.005 (0.994, 1.016)	1.001 (0.988, 1.014)
RUS	Male	0.63 (0.5, 0.8)^**^	0.61 (0.47, 0.79)^**^
	Female	0.92 (0.67, 1.27)	0.85 (0.54, 1.34)
	Total	0.77 (0.61, 0.97)^*^	0.72 (0.52, 1)^*^
RPS	Male	0.46 (0.3, 0.7)^**^	0.44 (0.29, 0.68)^**^
	Female	0.95 (0.5, 1.8)	0.91 (0.47, 1.76)
	Total	0.65 (0.42, 1.02)	0.63 (0.4, 1)
RMS	Male	0.47 (0.27, 0.81)^*^	0.48 (0.25, 0.95)^*^
	Female	0.79 (0.48, 1.31)	0.8 (0.48, 1.33)
	Total	0.62 (0.39, 1.004)	0.62 (0.37, 1.07)
RPM	Male	0.43 (0.2, 0.91)^*^	0.43 (0.22, 0.84)^*^
	Female	1.12 (0.36, 3.45)	0.91 (0.27, 3.07)
	Total	0.64 (0.3, 1.38)	0.62 (0.25, 1.58)

* *P* < 0.05,

** *P* < 0.01.

Model 1: Unadjusted covariates.

Model 2: Race was adjusted for TTFAT, TSFA, TMUFA, TPUFA, and RMS; Race, age, diabetes and hypertension were adjusted for RUS; hypertension, race and age were adjusted for RPS; Race and diabetes were adjusted for RPM.

**Table 3:** Subgroup analysis of association between fatty acids and NAFLD stratified by age and weight grades.

**OR (95% CI)**	**Subgroup**	**TTFAT**	**TSFA**	**TMUFA**	**TPUFA**
OR (95% CI)	18–36 Y	1.001 (0.996, 1.007)	1.008 (0.996, 1.021)	1.004 (0.99, 1.018)	0.994 (0.968, 1.02)
	37–54 Y	1.011 (1.004, 1.018)^**^	1.043 (1.021, 1.065)^**^	1.024 (1.003, 1.045)^*^	1.021 (0.998, 1.044)
	55–66 Y	1.002 (0.997, 1.006)	1.011 (0.995, 1.027)	1.004 (0.993, 1.016)	0.995 (0.977, 1.013)
	67–80 Y	1.004 (0.998, 1.01)	1.006 (0.99, 1.022)	1.009 (0.99, 1.028)	1.02 (0.996, 1.048)
OR (95% CI)	N-weight	1.006 (0.995, 1.016)	1.015 (0.985, 1.046)	1.018 (0.989, 1.047)	1.014 (0.981, 1.049)
	Overweight	1 (0.995, 1.004)	1.004 (0.992, 1.017)	0.997 (0.983, 1.011)	0.993 (0.97, 1.016)
	Obesity	1.007 (1.002, 1.011)^*^	1.025 (1.012, 1.038)^**^	1.014 (1.004, 1.025)^*^	1.008 (0.99, 1.027)

N-weight: normal weight. Weighted univariate logistic regression was used for analysis.

* *P* < 0.05,

** *P* < 0.01.

**Table 4:** Comparison of dietary fatty acids and ratios between sexes.

**Item**	**Male**	**Female**
TTFAT^***^	96.7 (1.5)	72.2 (1.6)
TSFA^***^	31.2 (0.6)	23.4 (0.6)
TMUFA^***^	33.6 (0.6)	24.7 (0.6)
TPUFA^***^	22.5 (0.4)	17.2 (0.4)
RPS	0.8 (0)	0.8 (0)
RPM^*^	0.7 (0)	0.7 (0)
RMS	1.1 (0)	1.1 (0)
RUS	1.9 (0)	1.9 (0)

Survey-weighted linear regression was used to calculate *P* values of continuous variables.

* *P* < 0.05,

*** *P* < 0.001.

**Figure 3:**
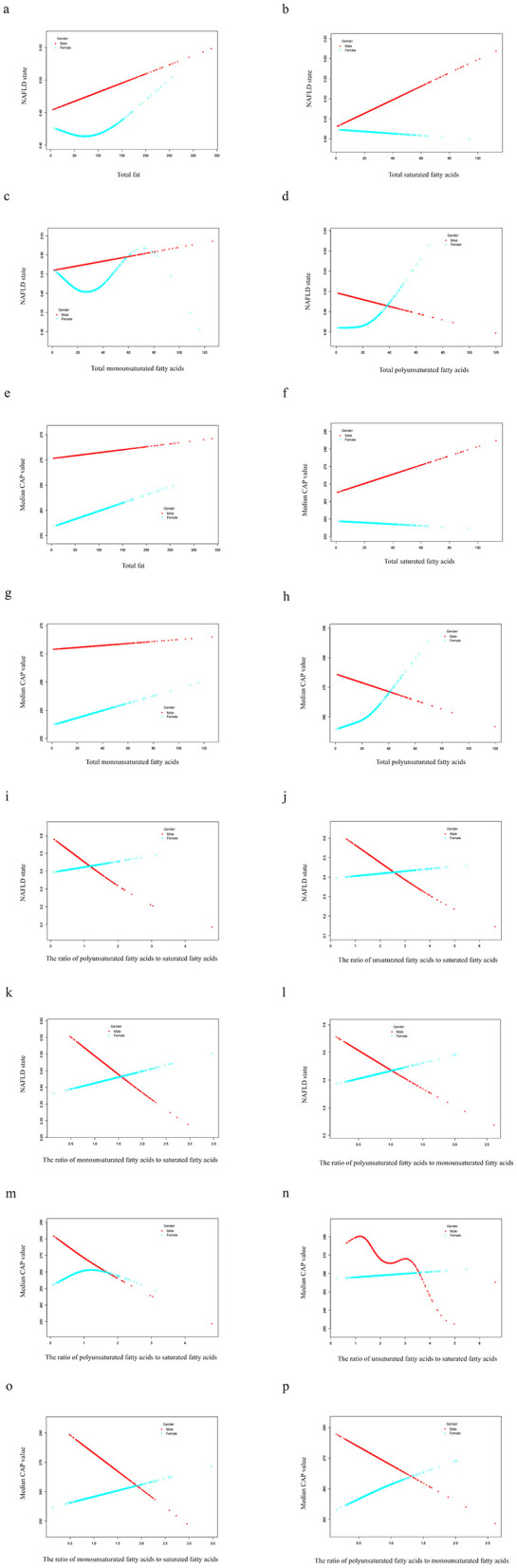
Smooth curve fittings. Generalized additive models were stratified by sex and adjusted for corresponding confounders. The dotted red line represents the smooth curve fit for males while the green line is for females. NAFLD, nonalcoholic fatty liver disease; CAP, controlled attenuation parameter.

**Figure 4:**
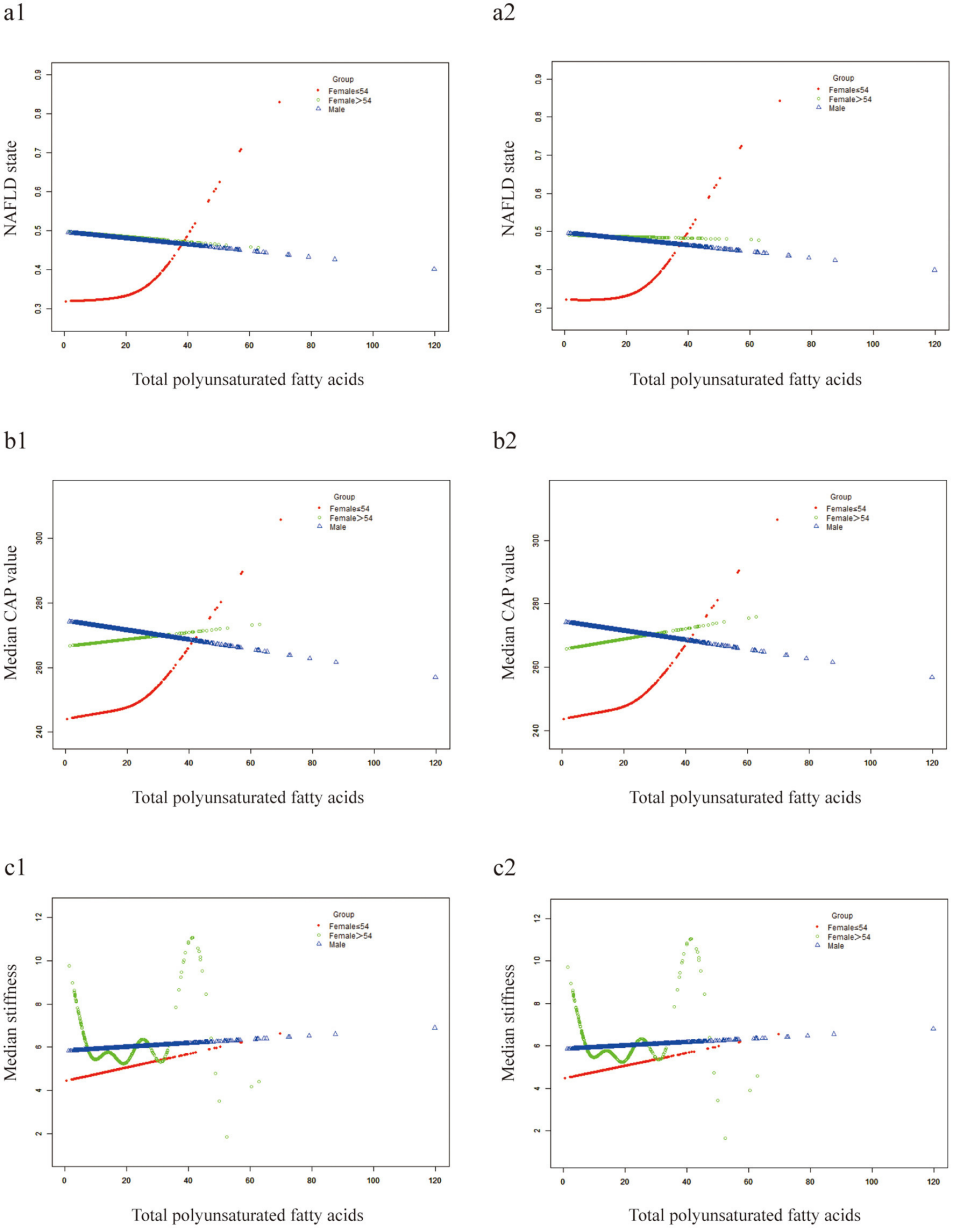
Smooth curve fittings. Generalized additive models were stratified by three groups. **(a1-c1)** Not adjusted for confounders; **(a2–c2)** adjusted for sex and race.

Smooth curve fitting exhibited a linear correlation between dietary fatty acids and NAFLD risk in males, whereas in females, the relationship is non-linear (Figure 3). Except for the TPUFA intake (Figure 3d), dietary fatty acids were positively correlated with the prevalence of NAFLD in males (Figures 3a–c). The slope of TMUFA was smaller than those of TTFAT and TSFA. Similar to the above trend, TPUFA presented a linear negative relationship with the steatosis severity of NAFLD in males as measured by CAP values, while all the other fatty acids displayed positive associations (Figures 3e–h). PUFA showed a protective effect against NAFLD in men, but the opposite was true in women. We hypothesized that sex hormones may play a role, so we conducted additional research. We divided the study population into three groups, women under 54 years of age, women over 54 and a male group. Smooth curve fitting was plotted for the relationship between PUFA and NAFLD stratified by aforementioned three groups. And the result was just as we expected (Figure 4). That is the curve for the women's group over 54 years of age almost overlapped with the curve for the men's group (Figure 4a1), suggesting that the protective effect of PUFA against NAFLD is similar in postmenopausal women and men. With regard to the fatty acid ratios, all showed negative associations with NAFLD in males but positive correlations in females (Figures 3i–l). As depicted in Figures 3i–l, all the curves were linear and the slope was greater in males than in females. As for the smooth curve fittings between ratios and CAP value, the correlations remained inverse between males and females (Figures 3m–p). A wave-shaped declining trend was observed between RUS and CAP value in males while other relationship curves appeared linear, with steeper slopes in males compared to females.

## Discussion

Our study confirmed that saturated fatty acid is a risk factor for NAFLD, consistent with existing literature. Saturated fat intake could contribute to increased intrahepatic triglyceride independent of total calorie intake (7). Excess intake of saturated fatty acids can accelerate the development of NAFLD via endoplasmic reticulum stress (23). Based on our study, we found that dietary lipid components can generally increase the risk of NAFLD except for PFUA. Furthermore, univariate logistic regression stratified by age stage and weight grade and age stage demonstrated that this association was significant only in participants aged 37–55 and in those who were obese. Compared with saturated fatty acid, unsaturated fatty acids presented relatively protective effects on NAFLD. Among the unsaturated fatty acids, PUFA is less likely to cause NAFLD than MUFA, coinciding with the conclusions derived from a recent study (24). *In vitro* experiments showed that saturated fatty acids induced significant cellular lipotoxic damage which was combated by monounsaturated fatty acids and polyunsaturated fatty acids (25). Diet may also influence NAFLD incidence by altering gut flora (6). As one of the polyunsaturated fatty acids, eicosapentaenoic acid can significantly reduce the level of triglyceride in the liver of UCP1 knockout and high-fat fed male mice, which was not observed in females (26). Previous research found a lower incidence of NAFLD in women than in men, but the disease develops more rapidly in postmenopausal women, suggesting a possible hepatoprotective role for estrogen (27). However, androgens may exhibit the opposite effect (28, 29). The smooth curve fitting in our study indicates sex-specific effects of dietary PUFA on NAFLD which we hypothesize that sex hormone may play a role. This finding diverges from Jiajia Cui's study. Thus, we conducted complementary analysis which proves postmenopausal women are as sensitive as men to the protective effects of PUFA against NAFLD. Animal study showed that estrogen activated antioxidant enzymes such as Sod2/Gpx1 through modulation of the signaling pathway consisting of the estrogen receptor alpha and peroxisome proliferator-activated receptor-gamma coactivator 1 alpha, and inhibited mitochondrial oxidative damage and inflammatory responses, resulting in female mice being more insensitive to high fat/high fructose diet-induced NAFLD (30), which partly explains the mechanisms underlying the sex-specific role of dietary components on the risk of developing NAFLD. Furthermore, our study also support the hypothesis proposed by Christopher et al., which is an increased ratio of saturated-to-unsaturated fatty acids, whether stored within or delivered to the liver, may play a role in the progression from simple steatosis to NASH (31). However, our study goes further by explore the role of sex in the relationship. Those ratios, including RUS, RPS, RMS, and RPM, appeared to affect NAFLD risk specifically in males, with higher ratios predicting a lower risk. The relationship remained significant even after adjusting for confounding factors. Previous reviews noted that while the role of MUFAs may vary across human and various murine models, PUFAs consistently exhibit anti-obesity, anti-steatotic, and anti-inflammatory effects (32), providing strong evidences for the definite protective effect of PUFA on NAFLD. A retrospective study based on the British Biobank showed that “PUFA enriched vegetarian” dietary pattern had therapeutic effect on NAFLD, while a “PUFA enriched carnivore” dietary pattern didn't increase the risk of NAFLD (33). The prevalence and severity of NAFLD, as well as major risk factors, present significant differences between sexes at different age stage (34). During the reproductive age, the prevalence and severity of NAFLD are higher in men than in women. However, the situation reverses after menopause, suggesting that estrogen is protective (27, 34). Our study also verifies the scientific validity of this conclusion. Several experimental studies attempted to explore the role of gender in the effects of dietary fatty acids on the development of NAFLD. As one of the polyunsaturated fatty acids, eicosapentaenoic acid can significantly reduce the level of triglyceride in the liver of UCP1 knockout and high-fat fed male mice, which was not observed in females (26). Cd36 is a fatty acid transporter protein. *In vitro* experiment showed that male mice had higher expression of the lipid uptake gene Cd36, which partly explains why males are more sensitive to dietary fatty acid-induced NAFLD (35). Ministrini et al. emphasized that sexual hormones controlled complex crosstalk of pro-inflammatory signals and prosteatotic signals mediated by Kupffer cells, lipid and glucose toxicity (36) which may account for the sex-specific influence of dietary fatty acids on NAFLD to some degree. Based on statistical results in Table 3, although PUFA did not contribute to the development of NAFLD in individuals of any weight class at any age, other fatty acids (TTFAT, TSFA, and TMUFA) significantly increased the risk of NAFLD in obese individuals. It was well described that adipocytes supplied more than two-thirds of fatty acids for hepatic triglyceride synthesis. And excess fatty acids not only induce hepatic insulin resistance but also impair insulin clearance which is proportional to the amount of liver fat in NAFLD (37). Thus, we surmise that the obese individuals are more likely to suffer from overload of fatty acids via intake of various fatty acid which may induce hepatic insulin resistance and impaired insulin clearance. In addition, an experimental study demonstrated that obese individuals have higher expression of NLRP3 which could promote the development of NAFLD under conditions of energy excess (38).

Our study compared the impacts of various fatty acids and related ratios on NAFLD based on large-scale real-world data. So far, few clinical studies have explored the confounding effect of sex and age on the relationship between dietary fatty acid intake and NAFLD. Notably, we explored the influence of sex, weight, and age on the correlation between dietary lipid and NAFLD and the findings are novel and reliable. However, there are some limitations in our study. This is a retrospective study in which selection bias, missing data, and residual confounding effects were inevitable. Besides, although the methodology was appropriate, it lacks innovation. Thus, further prospective randomized controlled studies, experimental studies and mass spectrometry analysis for global lipidomics are required to verify our conclusions and explore the potential cause-effect relationship. The role of sex-determined genetic susceptibility in the effect of fatty acids on the pathogenesis of NAFLD deserves further investigation.

## Conclusion

Overall, dietary fatty acids, except for polyunsaturated fatty acids, can promote the development of NAFLD, especially for obese individuals aged 37–54 years old. The associations between fatty acid ratios (RUS, RPS, RMS, and RPM) and NAFLD are sex-specific, displaying opposite trends between males and females. They have a higher predictive value of NAFLD in males than females, and the relatively protective effect of unsaturated fatty acids against NAFLD was observed exclusively in males. Polyunsaturated fatty acids have a higher protective value than monounsaturated fatty acids for NAFLD. Therefore, the dietary structure adjustment aimed at increasing the ratio of polyunsaturated or monounsaturated fatty acids to saturated fatty acids could be a promising strategy to prevent NAFLD and alleviate steatosis in males. Based on the current retrospective analysis, we suggested that the most important strategy to prevent NAFLD appears to be limiting overall dietary fatty acids intake, followed by increasing the proportion of polyunsaturated fatty acids in the diet, especially for middle-aged obese men.

## Data Availability

Publicly available datasets were analyzed in this study. This data can be found here: the datasets generated and/or analyzed in the current study are available in the NHANES database (https://wwwn.cdc.gov/nchs/nhanes/search/datapage.aspx?Component=Dietary&CycleBeginYear=2017).
